# Feasibility and Acceptability of an Interactive Mental Well-Being Intervention for People With Intellectual Disabilities: Pilot Mixed Methods Study

**DOI:** 10.2196/15190

**Published:** 2019-11-14

**Authors:** Leen Vereenooghe, Kristian Westermann

**Affiliations:** 1 Faculty of Psychology and Sports Science Bielefeld University Bielefeld Germany

**Keywords:** intellectual disabilities, feasibility studies, tablet computer, mental health

## Abstract

**Background:**

The availability of both digital and traditional mental well-being interventions is rising, but these interventions typically do not consider people with intellectual disabilities as potential users.

**Objective:**

The study aimed to explore the acceptability and feasibility of a new digital intervention, developed with and for people with intellectual disabilities, to improve their subjective well-being.

**Methods:**

Using a single-group pre-post design, participants with intellectual disabilities and their caregivers completed the 4-week intervention. Mixed methods questionnaires assessed the acceptability of the intervention, in addition to self-report and proxy-report measures of subjective well-being and behavioral problems.

**Results:**

A total of 12 men with mild to moderate intellectual disabilities enrolled in and completed the study alongside 8 caregivers. Participant acceptability of the intervention was high, and feedback covered multiple aspects of the intervention, including (1) program concept and design, (2) program content, and (3) intervention usage. Self-rated mood barometers indicated mood improvements for 5 participants, deteriorations for 2 participants, and no observed changes for the remaining participants. Statistical analyses yielded no difference from pretest (median=79; range 39-86) to posttest (median=79; range 21-96) for subjective well-being in people with intellectual disabilities (W=10.5; *P*=.17) and for behavioral problems (W=14; *P*=.05).

**Conclusions:**

People with intellectual disabilities and their caregivers are receptive to using digital well-being interventions, and this research shows such interventions to be feasible in routine practice. Given the acceptability of the intervention, its potential efficacy can now be evaluated in people with intellectual disabilities and symptoms of reduced mental well-being.

## Introduction

### Background

People with intellectual disabilities have an increased vulnerability for developing mental health problems, in part owing to the frequency of negative life events and experiences that are common in this population, for example, experiencing stigma, isolation, dependency on caregivers, and experiences of abuse [1-3]. Although they constitute a heterogeneous population, people with intellectual disabilities generally present with a wide range of difficulties in communication, cognitive, and adaptive skills with age of onset before 18 years. These difficulties can then act as an additional burden on their ability to cope with such negative life events, thereby increasing their risk of poor mental health.

There is comparably little research and clinical attention for this population, in spite of their vulnerability and the resulting high prevalence of mental disorders. In terms of treatment or intervention options, pharmacological therapies are available, but also frequently prescribed in the absence of diagnosed mental disorders [4]. Psychological therapies offer a viable alternative, with cognitive behavioral therapies (CBT), in particular, appearing very promising in their treatment of anxiety, aggression, and mood disorders [5,6]. Unfortunately, therapeutic disdain for working with this population as well as diagnostic overshadowing and misdiagnoses may contribute to the slow uptake of therapists to offer psychological therapies to this population [7-9]. Meanwhile, the assumption that people with intellectual disabilities lack the cognitive skills to undertake therapy has been challenged, in particular with regard to CBT [10,11]. Although people with mild intellectual disabilities and better verbal and communicative abilities are more likely to understand the CBT framework and concepts of cognitive mediation [12], certain CBT skills can be trained using computerized or video-based training paradigms in people with mild and moderate intellectual disabilities [13-15]. This paves the way for a third potential intervention strategy in the form of computerized or Web-based mental health interventions as an adjunct or preparatory step for traditional talking therapies and pharmacotherapies.

New technologies present a range of applications that people with intellectual disabilities can benefit from, from mobile apps to improve problem-solving skills to virtual and augmented reality applications for social, vocational and self-determination skills [16-18]. The use of computers in psychological therapy for people with intellectual disabilities is still uncommon, despite such interventions being widely available for a wide range of mental health problems to people without intellectual disabilities. To date, only one full computerized CBT intervention has been evaluated in the treatment of anxiety and depression in adults with intellectual disabilities [19]. Although the cost-effectiveness of this intervention has not been evaluated, its acceptability among both patients with intellectual disabilities and their treating therapists was high. This is in line with research showing that both groups are interested in introducing technology as an additional means to improve the mental health and the therapy process involving people with intellectual disabilities [20].

A key challenge in delivering internet interventions to people with intellectual disabilities is the accessibility of such interventions. Barriers include economic factors related to the cost of computers and difficulties associated with potential cognitive and physical limitations [21]. Turning to internet use, access rates in people with intellectual disabilities are much lower than that in people without intellectual disabilities [22]. Here, caregivers play an important role in providing people with intellectual disabilities access to technology and the internet, as their support is often required to obtain the devices and set up user accounts. They may also be inclined to protect people with intellectual disabilities from the risks associated with internet use, although this can result in people with intellectual disabilities missing the benefits of internet use in relation to self-efficacy, empowerment, and social networking [23].

### Objectives

Taking together the vulnerability of people with intellectual disabilities to experience mental health problems and the potential of digital interventions as an addition to traditional treatment interventions, this study presents an initiative to provide an accessible digital intervention to promote the mental well-being of people with mild to moderate intellectual disabilities. The driving objective is that successful mental health promotion might present an alternative and preventative strategy to improve the mental well-being of people with intellectual disabilities, in addition to or before the treatment of mental health problems through established pharmacological or psychotherapeutic interventions. To this extent, we set out to develop an intervention that fulfilled the following criteria:

1. Including people with intellectual disabilities in the development and design phases of the intervention to ensure its accessibility and its acceptance by people with intellectual disabilities.
2. Incorporating cognitive-behavioral components from evidence-based interventions.
3. Adopting a resource-oriented approach which involves the caregivers and support workers to assist with the implementation of the intervention in daily life.
4. Offering content that is personally relevant or can be customized.

In this paper, we briefly described the development of the intervention and presented a small pilot study to explore its potential feasibility and practicality as well as gave a first indication of its potential efficacy.

## Methods

### Phase I: Intervention Development

The intervention was intended to provide tips to improve the users’ psychological well-being. Program content was derived from existing empirically tested programs and included both psychoeducational, behavioral activation and cognitive restructuring components. An advisory group of people with and without intellectual disabilities was established to provide feedback during the development process.

#### Content Development

The intervention comprised 8 modules which covered the following topics: (1) participation, (2) being active, (3) friendships, (4) relaxation, (5) self-acceptance, (6) communication, (7) self-actualization, and (8) cognitive restructuring (Textbox 1 provides an overview of the module aims).

**Outline and modules of the Pudelwohl intervention.**

Module 1: Participation.

- Aim: Understanding that participation may lead to improved social contact and social support as well as being enjoyable.

Module 2: Being active.

- Aim: Improving mental well-being through increasing physical activity.

Module 3: Friendships.

- Aim: Highlighting the importance of a supportive network and tips for maintaining friendships.

Module 4: Relaxation.

- Aim: To acknowledge the need for relaxation and how this can be achieved.

Module 5: Self-acceptance.

- Aim: Addressing and accepting your strengths and weaknesses, including your disabilities.

Module 6: Communication.

- Aim: Being able to communicate your needs using a positive communication style.

Module 7: Self-actualization.

- Aim: Behavioral activation and finding of activities that support your personal development.

Module 8: Cognitive restructuring.

- Aim: Identifying and changing helpful and unhelpful thoughts to help change how we may feel about a situation.

Modules were spread over 4 sessions to be held at 1-week intervals. The first session started with an interactive introduction to learning to use the tablet, followed by module 1. Sessions 2 and 3 comprised 3 modules each. The fourth and final session presented module 8, followed by a reflection on all past modules and the instruction to choose which 3 tips they considered to be most important to them. These top 3 were then transferred onto a paper certificate, which participants received at the end of the study and acted as a self-help reminder. Furthermore, sessions 1 to 3 ended with a brief summary and homework assignment, whereas sessions 2 to 4 started with a reflection on the last session and a brief evaluation of the chosen homework assignment.

#### Technical Features

Adobe Captivate (2017, Version 10.0.0.192) was used to develop the intervention. The intervention was designed for use with 11.6-inch Odys Primo Win 12 2-in-1 tablet computers, which would be lent to the participants in the study. The images used within the intervention were either self-generated or license-free materials.

The intervention was introduced and led by an animal avatar: a poodle named *Wohl*, which references a German wordplay involving well-being. This poodle avatar explains how to navigate through the slides using the on-screen arrows and what users can expect from the program. This includes explaining symbols used throughout the program that introduce quizzes and discussions. Some quizzes prompt users to evaluate statements as helpful or unhelpful or good or bad, whereas in other quizzes users are given multiple-choice questions using drag-and-drop selection methods. Discussion slides present a question or statement that the user is encouraged to respond to or discuss together with their support worker. The main poodle avatar also provides an overview of his 8 animal friends, represented by 8 different animal avatars, that are associated with each module.

Overall, we aimed to minimize visual distractions by refraining from presenting a table of contents or navigation menu. To enhance user engagement, slides were animated where possible and were provided both on-screen text and a voice-over.

#### Advisory Group

An advisory group was established in collaboration with a local information technology workshop for people with and without disabilities or mental disorders. Flyers were distributed at the workshop to inform their users about our study. Workshop staff confirmed the time and date of the 4 planned consultations with users who expressed an interest. The consultations were held at 2-week intervals, with attendance varying between 4 and 7 participants, of which at least 3 presented with intellectual and developmental disabilities. We did not employ specific inclusion or exclusion criteria for people to attend the consultations. As these attendees were not participants in a research study, we did not routinely collect information regarding their sociodemographic characteristics, but all advisory group members were adults, with more men than women attending the consultations. During each session, the researchers presented drafts of the program modules and requested feedback regarding module content, design, accessibility, interactivity, and delivery. Changes, as a result of this feedback process, were presented at consecutive meetings for further evaluation and included changing font sizes, simplifying language, increasing and simplifying program interactivity, changes to program navigation options and page layout, and the use of supportive instead of corrective feedback in exercises. Feedback that was not incorporated for the pilot study because of limited resources included the provision of male and female voice-overs, more animations, and mutable background music.

A more detailed description of the co-development process for the intervention is given by Vereenooghe and Westermann [24].

### Phase II: Pilot Study

#### Recruitment

People with intellectual disabilities and their support workers from 2 residential facilities in North Rhine-Westphalia, Germany, took part in the study. Both facilities provide services for children and young adults with intellectual and developmental disabilities. Support staff were informed about the study objectives and procedures and were asked to identify potential participants with intellectual disabilities among the residents whom they supported.

We aimed for minimal exclusion criteria to reflect the heterogeneity found within the population of people with intellectual disabilities and only excluded participants who (1) had a diagnosis of autism spectrum disorders without intellectual disabilities and (2) people with severe or profound intellectual disabilities who might not be able to operate the tablets and engage with the program content. Details regarding participants’ level of intellectual disabilities was obtained by the participating support workers from the service users’ records.

The sole inclusion criterion for support workers was the requirement of a minimum of 2 contact hours per week with the participant with intellectual disabilities to ensure both participants had sufficient time together to implement the intervention.

#### Design

A single-group pre-post design was used, with no control condition. Outcomes of mental well-being were assessed at pretest and posttest, in addition to mood barometers completed before each session. No follow-up data were obtained for this pilot study, for which recruitment took place from March 2018 to June 2018.

#### Measures

##### Primary Outcome

Program evaluation questionnaires were completed at posttest to assess the acceptability of the intervention. Questions were derived from the Client Change Interview Schedule [25] as implemented by Earley et al [26] and used both forced-choice and open-ended response formats. People with intellectual disabilities used a picture-based Likert scale to communicate their feedback regarding the program, similar to that used in the other outcome measures. The questionnaires were administered by the second author KW during a meeting organized with the participant with an intellectual disability after their support worker had indicated that they had completed all sessions. Posttest assessment took place within 2 weeks following the last session.

Questions for participants with intellectual disabilities included “what in particular do you still remember about the intervention?” and “which aspects did you like?” Support workers’ questionnaires asked whether they had noticed any change in the well-being of the person with an intellectual disability. Their version also asked more explicitly to identify specific content they considered to be helpful or not and what negative and positive aspects they have kept in mind.

##### Secondary Outcomes

The Personal Well-Being Index—Intellectual Disability (PWI-ID) [27] was translated to German based on the German version of the PWI [28] and administered to participants with an intellectual disability by their support worker. Administration took place within 1 week before the first session and within 2 weeks following the last session. The 8-item measure asks how people feel about their material, physical, mental, and general well-being. Responses are given on a Likert scale using 2, 3, or 5 facial expressions ranging from sad to happy and are then converted to numerical percent scores, with higher scores indicating better well-being. The number of response options (2, 3, or 5) is established for each participant before the assessment to optimize the validity of the responses.

Participants also completed mood barometers before each session, using the same picture-based Likert scale as used with the PWI-ID. To this extent, they received assistance from their support worker.

The Aberrant Behavior Checklist—Community (ABC-C) [29,30] was used as an indicator of informant-reported mental well-being, as it includes indicators of mental disorders in people with intellectual disabilities. Support workers rated the severity of the 58 behavioral problems over the last 4 weeks, using a 4-point Likert scale, with higher scores corresponding with more serious behavior problems. Support workers completed this measure independently following the instructions of the second author KW. The ABC-C was completed within 1 week before the first session and within 2 weeks following the last session.

#### Procedures

Participants were instructed to complete 1 session per week, with a maximum of 2 sessions per week. Tablets containing the program were lent to the participating organizations so people had instant access to the devices. The intervention was implemented in the regular working time of the support workers. Support workers had a facilitating role, and the intervention was intended to be used by the person with an intellectual disability as its primary user. Support workers could provide both practical and content support, for example, regarding the use of the tablet computers (eg, volume control or recharging the devices), clarifying the content (eg, repeating or reading the on-screen information), and assisting with program demands (eg, prompting the user to select an on-screen response or engaging the user in a discussion).

#### Ethical Concerns

Ethical approval for this study was obtained from the Bielefeld University Ethics Committee. All participants received information about the study in easy-to-read language and provided written informed consent. Participants without the capacity to provide informed consent were not enrolled in the study.

#### Data Analysis

Responses from the feedback questionnaires regarding the acceptability of the intervention were analyzed using quantitative content analysis of qualitative data [31]. First, all questionnaires were read to get an initial overview of the participant responses. Next, we generated codes to summarize each distinct piece of information, thereby being guided by the raw data rather than using pre-existing codes derived from the theory. For the qualitative analysis, we grouped these themes in overarching themes. For a further quantitative content analysis, the codes were then counted to identify themes that appeared more prevalent and to explore patterns in the data that could guide future development. Within this step, however, we made the decision to interpret the thematic codes as being positively or negatively valenced based on the questions that elicited these responses (eg, positively valenced questions included “which aspects did you like?” and “which content did you find helpful?” whereas negatively valenced questions, included “what did you notice negatively?” and “which content did you find unhelpful?”).

Differences in pretest and posttest scores were analyzed in SPSS (2017, Version 25) using nonparametric 1-tailed Wilcoxon signed rank tests.

## Results

### Participant Characteristics

**Figure 1:**
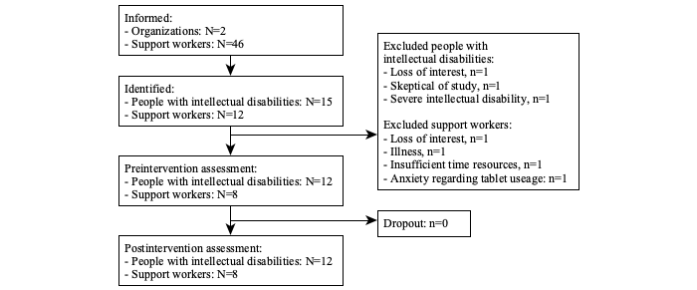
Study flowchart.

Following eligibility checks and informed consent procedures, 12 pairs of participants completed baseline assessments (Figure 1). A total of 3 support workers completed the study with multiple participants with intellectual disabilities. Participants with intellectual disabilities were all male, whereas support workers included 5 women and 3 men who had worked with the participant with intellectual disabilities for at least 6 months (Table 1).

### Intervention Feasibility, Acceptability, and Satisfaction

None of the enrolled participants, either people with intellectual disabilities or their support workers, dropped out during the study or withdrew their consent. The overall recruitment success (completed/informed) rates were 26% (12/46) for support workers and 80% (12/15) for people with intellectual disabilities (Figure 1). Owing to time constraints, 1 participant pair completed 2 sessions per week and completed the intervention in less than 4 weeks.

**Figure 2:**
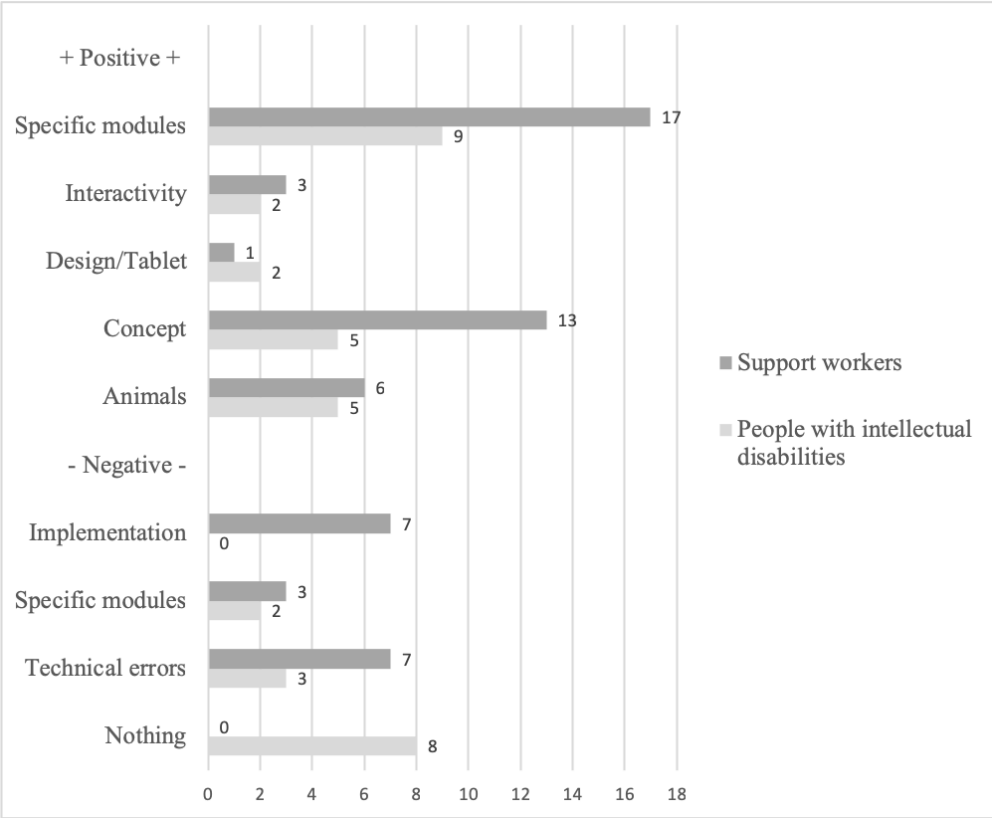
Acceptability of Pudelwohl as indicated by code frequency of the quantitative content analysis of the Program Evaluation Questionnaires.

Content analysis of the program evaluation questionnaires was classified into 3 main areas: (1) program concept and design, (2) program content, and (3) intervention usage. Analysis of the positive and negative feedback, as shown in the quantitative content analysis in Figure 2, provided support for the conceptualization of the Pudelwohl intervention and its specific modules. It also indicated desired improvements for the implementation of Pudelwohl.

**Table 1:** Sociodemographic characteristics of participants with intellectual disabilities (N=12) and support workers (N=8).

Sociodemographic characteristics, variable		Value
**People with intellectual disabilities**		
	Age (years), median (range)	19 (17-24)
	Sex, male, n (%)	12 (100)
	Mild intellectual disability, n (%)	9 (75)
	Moderate intellectual disability, n (%)	3 (25)
**Support workers**		
	Age (years), median (range)	42 (28-58)
	Sex, male, n (%)	3 (38)
	Years working with participant, median (range)	3 (0.5-6)

#### Program Concept and Design

The use of animal avatars was liked by the majority of people with intellectual disabilities and support workers. The interactive design, with quizzes, and use of both images and videos were also positives of the program. Support workers valued the program as a user-appropriate concept based on its audiovisual approach, the use of repetition, encouragement and supportive feedback, and session length. Given the heterogeneity of people with intellectual disabilities in terms of cognitive and communicative abilities, some support workers also rated the intervention as either too childish or difficult with regard to language. By contrast, none of the participants with intellectual disabilities mentioned the program to be childish and instead were explicitly positive about the design of the intervention.

#### Program Content

The friendships, self-acceptance, and self-actualization modules were particularly well received by people with intellectual disabilities, as were specific tips to improve your well-being. Support workers approved the wide range of topics and the information regarding local service providers included in specific modules and also highlighted the need to provide more concrete and less abstract tips in the self-actualization module. They considered the content of the modules concerning maintaining friendships, being active, and relaxation as most helpful to the people they were supporting. Both user groups considered the communication module too difficult, which is likely because of the metaphor used to explain the different roles and aspects of good communication (ie, the talker, the listener, a conversation topic, switching talking-listening roles, and changing topics).

#### Intervention Usage

Technical errors in the use of the program were a main hindrance for both people with intellectual disabilities and their support workers. This applied to navigation arrows that were considered too small and buttons or interactions which were inactive. Session length was appropriate, according to support workers, and implementation should be limited to 1 session per week.

Overall satisfaction rates for the 4-week intervention were high. Participants with intellectual disabilities and support workers gave the program approval rates of 92% and 86%, respectively.

### Evaluation Outcomes

**Figure 3:**
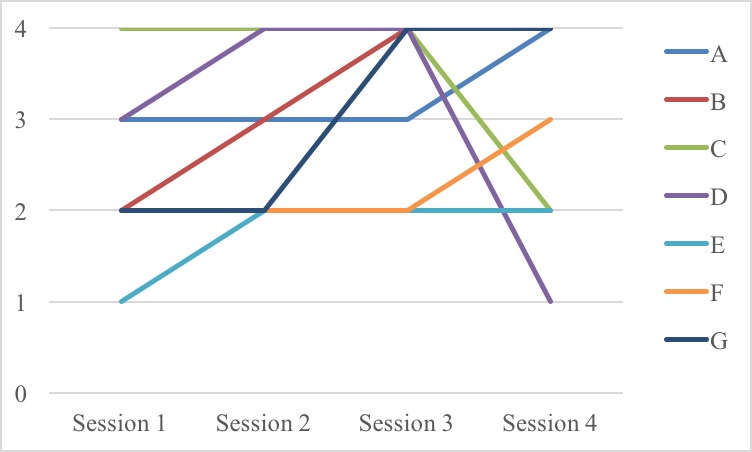
Mood ratings of participants with intellectual disabilities with fluctuating mood during the intervention (N=7).

The picture-based mood barometers for participants with intellectual disabilities administered before each session were converted to a 5-point Likert scale ranging from 0 to 4, with higher scores indicative of a better mood. Descriptive analysis showed improved moods for 5 participants and deteriorations for 2 participants (Figure 3). A total of 5 participants gave the maximum mood rating at each measurement point and are, therefore, not included in Figure 3.

Table 2 presents changes in self-reported personal well-being and proxy-report behavioral problems from pretest to posttest. Data are reported separately for the 2 organizations because of the observed differences in their baseline sample characteristics. Statistical analyses were conducted, as planned, with the full sample. PWI-ID data for 3 participants were excluded due to acquiescent responding. Wilcoxon signed rank tests yielded no statistical difference from pretest (median=79; range 39-86) to posttest (median=79; range 21-96) for subjective well-being in people with intellectual disabilities (W=10.5; *P*=.17). There was also a nonsignificant reduction in behavioral problems (W=14; *P*=.05) as reported by support workers at pretest (median=19; range 0-94) and posttest (median=14; range 1-73).

Finally, data from the program evaluation questionnaires showed that half of the support workers did not observe any changes in the behavior or well-being of the participants with intellectual disabilities. Other support workers did report an improved relationship with the participant with intellectual disabilities, as well as increased interests in daily activities associated with the program content (including planning activities, self-acceptance, and independent use of computers).

**Table 2:** Median and range for subjective well-being (Personal Well-Being Index—Intellectual Disability, N=9) and behavioral problems (Aberrant Behavior Checklist—Community, N=12) at baseline and post intervention.

Outcome measure		Baseline, median (range)	Post intervention, median (range)
**Personal Well-Being Index—Intellectual Disability**			
	Organization A (n=6)	79 (39-82)	79 (21-96)
	Organization B (n=3)	54 (54-86)	64 (57-79)
**Aberrant Behavior Checklist—Community**			
	Organization A (n=8)	29 (10-94)	21 (11-73)
	Organization B (n=4)	4 (0-10)	3 (1-3)

## Discussion

### Principal Findings

This pilot study shows that it is feasible to develop a digital psychoeducational intervention—with multiple brief sessions of interactive content—for use in routine practice. We also observed initial improvements in subjective well-being and reduced behavioral problems in some participants. Participants’ interest in the study and the absence of any dropouts further indicate that an evaluation of the intervention’s efficacy in a larger sample would be feasible.

The participatory approach in developing the intervention together with people with and without intellectual disabilities or mental health problems was both necessary and valuable in the changes that were made to the program. User-driven changes sometimes contradicted universal design recommendations that were otherwise expected to improve the accessibility of digital materials [21]. Their involvement in the intervention design is likely to have positively impacted upon the eventual uptake and acceptability of the intervention in this pilot study. The discrepancy between people with intellectual disabilities liking the use of animal avatars and other images and some support workers considering the intervention to be childish further highlights the need to involve all end users in the design of an intervention and not be satisfied with the input from only support workers or people with intellectual disabilities.

### Study Limitations and Strengths

Key limitations of this study are the lack of randomization procedures and follow-up measures as well as the small sample size. These were deliberate decisions as the main objective was not to statistically evaluate the intervention’s efficacy but to investigate, using a naturalistic study design, whether it would be acceptable and feasible to implement the proposed intervention in the weekly routine of people with intellectual disabilities, and whether the recruitment of people with intellectual disabilities for an interventions study would be feasible.

The absence of any statistical effects of improved mood and behavior is also not surprising as we did not employ a threshold score on either of the outcome measures to select a sample where improvements in the mental well-being and behavioral problems were most desirable.

We also noted that the 4-week duration of the study is likely to have been too short for participants to implement meaningful changes and to record their potential impact. Meanwhile, the quick succession of very brief modules on distinct topics did not allow for a comprehensive or thorough work-through for each module. Hence, participants may not have had sufficient time to explore and master the attitudes, knowledge, and skills related to each module. Although from a clinical perspective that would have been more useful, this study focused primarily on exploring whether such content could be delivered using a digital format. The brief modules in combination with the qualitative feedback, therefore, enabled us to identify which modules in particular could be considered more useful for expansion in the intervention’s further development.

In spite of our efforts to recruit a representative sample, the final sample of people with intellectual disabilities was all male. It is not clear whether this reflected a bias by the support workers in identifying potentially interested participants, or an actual gender difference in interest to participate. Previous studies indicate, however, that technology use among people with intellectual disabilities is generally higher for adolescents and young adults aged below 30 years of age and for men [32,33].

The technical errors, as mentioned by the participants, were an additional negative aspect of the intervention. In spite of this, the overall approval ratings were nevertheless high.

Finally, although no participants dropped out during the study, it is unclear to what extent they adhered to the intervention contents and completed the homework tasks as we did not collect any data on actual use of the intervention. Given that usage characteristics may not be the ideal means of assessing adherence for electronic health technologies [34], future studies could explore to what extent participants experimented, tried, or internalized specific contents.

### Future Research and Clinical Implications

#### Overview

The Pudelwohl intervention, in its content and design, most closely approaches a combination of the 2 intervention arms of the Beat It trial, behavioral activation and guided self-help, both of which led to patient improvement [35]. Direct comparison of both studies demonstrates that the pilot Pudelwohl intervention resulted in higher completion rates (95% vs 81%), but also yielded lower effect sizes as our study design was not set up for evaluating intervention efficacy. A larger trial of the present intervention in a similar sample of people with at least subclinical symptoms of reduced well-being is required to more adequately compare both the interventions. A shared finding, however, is that participants with intellectual disabilities in both studies reported a desire for more sessions or an overall longer intervention [36].

Focusing on the digital components of our intervention, we found similar levels of acceptability to that of a website aimed at informing people with intellectual disabilities about good mental health [37]*.*

In terms of customizability and tailoring the intervention to individual participant needs, our intervention offers a compromise between a more rigid design of a website and the flexibility that can be offered in a face-to-face therapy, for example, by presenting options for local activities, giving participants the option to choose those tasks that are more relevant to them, and by involving support workers who could ensure program content was discussed in a way that was meaningful to the participant. However, it is not yet clear whether the acceptability and efficacy of individually tailored digital interventions is higher than that of standard packages [38].

#### Adapting Digital Interventions for People With Intellectual Disabilities

Contributing to the intervention’s overall acceptability is our purposeful attempt to adapt all aspects of the intervention to the variable needs of people with intellectual disabilities. This includes incorporating all but one of the suggested adaptations to psychological therapies for people with intellectual disabilities, as put forward by Hurley et al [39]. Use of a digital platform should not exempt the intervention from addressing the users’ intellectual disability; however, this disability in itself may present the person with considerable challenges and negative events in their daily lives that could contribute to poor mental well-being. In our intervention, we addressed this in Module 5 *Self-acceptance*, where participants were prompted to both say what they are good at and to reflect on questions such as *my weaknesses are not failures*, *you can laugh about some mistakes*, *I should feel bad about my weaknesses*, and *my weaknesses are a part of me*.

#### Defining the User Group: Should We Include Support Workers?

Involving support workers is generally viewed favorably when planning or delivering psychological interventions to people with intellectual disabilities [35,39,40]. When using digital interventions, their involvement can help overcome technical difficulties experienced by people with intellectual disabilities, as we found in this study. By contrast, their involvement could also present an extra barrier when support workers do not feel sufficiently confident themselves in using technologies or wish to protect the person from the potential risks associated with the use of Web-based technologies [23]. This approach could therefore lead to people with intellectual disabilities who might otherwise express an interest in digital interventions or who would have the capacity and skills to take part without the need for a support worker to be excluded from research or interventions built around the involvement of these gatekeepers. In this study, the challenges arising from such a design are apparent from the contrast between the low recruitment rate of support workers and the high interest of people with intellectual disabilities.

When a decision is made to include support workers, either as active participants or in a supporting role, attention should also be directed to any potential effects of the intervention on the support worker and their relationship with the person with an intellectual disability. This could include changes in attitudes, knowledge, or skills, as well as the quality of the relationship, which was reported to have improved by some of the support workers in this study.

### Conclusions

Overall, and in spite of the methodological shortcomings of this pilot study, this study clearly shows that internet interventions to improve the mental well-being of people with intellectual disabilities are worth further exploring as an additional intervention or prevention strategy, alongside more traditional psychopharmacological and psychotherapeutic approaches. Involving people with intellectual disabilities in the design of the intervention and ensuring the involvement of support workers and personal relevance of the intervention contents were major contributors toward the acceptance and feasibility of this intervention. The higher interest and usage rates of digital technologies in young people with intellectual disabilities identify them as the likely initial target group for such interventions.

## Abbreviations

ABC-C: Aberrant Behavior Checklist—Community
CBT: cognitive behavioral therapies
PWI-ID: Personal Well-Being Index—Intellectual Disability
